# Levamisole Usage as an Adjuvant to Hepatitis B Vaccine in Hemodialysis Patients, Yes or No?

**DOI:** 10.5812/numonthly.3985

**Published:** 2012-12-15

**Authors:** Houshang Sanadgol

**Affiliations:** 1 Department of Nephrology, Faculty of Medicine, Zahedan Medical University, Zahedan, IR Iran

**Keywords:** Hepatitis B, Vaccination, Hemodialysis Units, Hospital

## Abstract

**Background:**

Hepatitis B virus (HBV) infection is much more common in hemodialysis patients than the general population. These patients have an impaired immune response to HBV vaccination; to that end there are certain studies that have evaluated levamisole as an immunomodulator agent improving HBV vaccination response rate in hemodialysis patients.

**Objectives:**

In the current review, we have assembled all of the results to determine whether lavamisole is of value as an adjuvant to HBV vaccination in hemodialysis patients.

**Materials and Methods:**

Science Direct (Elsevier), ProQuest, Springer, MD Consult, BMJ Journals, Pubmed and Wiley were searched for levamisole application to HBV vaccination in hemodialysis patients. All studies revealed a seroconversion response level between levamisole plus HBV vaccine versus HBV vaccine alone.

**Results:**

From 10 relevant studies, 5 studies fulfilled our inclusion criteria. Three of them suggested the significant benefit of adding levamisole to the HBV vaccine to increase augment seroprotection level in hemodialysis patients. Another study reported a decrease in seroprotection level and another study showed no significant difference caused by levamisole administration.

**Conclusions:**

Due to the limited number of studies evaluated, it is challenging to perform a definite decision about routinely administering levamisole in addition to the HBV vaccine for all hemodialysis patients. However, it does seem reasonable to recommend administration of levamisole for impaired immune response patients.

## 1. Background

Hepatitis B virus (HBV) infection is a major global health issue which affects more than 2 billion people worldwide (1). Approximately 2 million people die from this infection every year (2, 3). Hemodialysis patients are at high risk for HBV infection due to the dialysis process and prolonged blood exposure in extracorporeal circuit (4). Although there are extensive control precautions and prevention measures in dialysis centers, all dialysis patients, staff members and end-stage or pre end-stage renal disease patients should be vaccinated against HBV which is still the most effective way to prevent the disease (5-10). Hemodialysis patients have an impaired cellular and humeral immune responses to HBV vaccination which is due to uremia-associated immunodeficiency such as lymphopenia with diminished T4 and T8 lymphocytes ,T-cell CD4 dysfunction, less IL-2 and IgG production from peripheral blood lymphocytes (11-15). Moreover after completion of the vaccination program, the antibody titer of responder hemodialysis patients is low and falls rapidly (16-19). Therefore, various strategies for improvement of the immune response have been formulated to increase the HBV vaccination response in hemodialysis patients such as changes in vaccination method, co-administration of zinc, γ-interferon, granulocyte-macrophage colony-stimulating factor (GM-CSF), and levamisole. These measures are all used to enhance seroconversion after HBV vaccination (10). However, the majority of these strategies are still experimental and not recommended as routine procedure.

Levamisole (LMS), a synthetic and soluble phenylimidazothiazole, has been widely used as a pesticide for domestic animals for more than 50 years (20). Nonetheless, it has been used in many treatment protocols for different purposes since 1972 (as it was discovered having immunomodulatory role as well as anticancer effect) (21-29). Levamisole increases natural killer cells, enhances B lymphocyte function, stimulates depressed T-cell activity and improves chemotaxis of granulocytes (30-32). Many studies have evaluated the effect of adding lavamisole as an adjuvant to HBV vaccination in hemodyalisis patients, but have had many conflicting outcomes.

## 2. Objectives

In our study we reviewed all these different results and with a view to sanctioning or not sanctioning lavamisole administration as an adjuvant to HBV vaccination in hemodialysis patients. The main point of interest is to evaluate the seroconversion response level to HBV vaccination in the study (HBV vaccination plus levamisole) and in control groups (HBV vaccination alone). Patients with serum antibody level more than 10 mIU/mL were considered as responders and the decision was made as to whether levamisole should be added as an adjuvant to HBVvaccination program in hemodialysis patients.

## 3. Materials and Methods

### 3.1. Search Strategy and Data Extraction

In this paper we searched all available reliable electronic and published sources using Science Direct (Elsevier), ProQuest, Springer, MD Consult, BMJ Journals, Pubmed and Wiley. The key words ‘levamisole’, ‘Hepatitis B Vaccination’, ‘Hemodialysis’ and ‘Vaccine adjuvant’ were used. We included both invivo and invitro studies that were published in English and non-English from Jan. 1980 to Nov. 2011. A single investigator (M.K) extracted all relevant data and inserted them into an electronic database that was checked twice. All of the studies reviewed and used in this paper have been agreed upon by all authors.

### 3.2. Inclusion / Exclusion Criteria

Inclusion criteria were the following:

- Studies that compared the response level between levamisole plus HBV vaccination vs. HBV vaccination alone

- Patients on maintenance hemodialysis

- Studies based on plasma-derived or recombinant DNA hepatitis B vaccination

- Studies that described dose schedules and route of vaccination

- Seroprotection response level reported at least 1 month after the last vaccination dosage

- Studies that enrolled adult patients

- Controlled clinical trials/studies that provided both case and control groups

Exclusion criteria were as the following:

- Patients with positive HBs Ag serology

- Patients with hepatitis B surface antibody (HBs Ab) titer higher than 10 mIU/M [responder patients]

- Patients with a positive history of malignancy

- Receiving immunosuppressive drugs 

- Patients with a positive human immunodeficiency virus antibody test (HIV Ab) or positive hepatitis C virus antibody (HCV Ab)

- Studies that enrolled child patients

- Studies that only published as abstracts or review articles 

Naive patients (who entered into primary vaccination program) and unresponsive patients (who do not respond to the vaccination program) were also eligible for our study.

## 4. Results

Among 604 citations, we identified 10 relevant studies in the literature review. One of the studies was excluded because it included child individuals (33). Another excluded case was positive HBs Ag serology (34). Two review articles were also excluded (35, 36). In addition, a study was excluded since the authors did not define the response rate of levamisole group (37). As shown in Table 1 and Table 2 , we obtained 5 studies that fulfilled our inclusion criteria and reported seroconversion response rates between levamisole plus HBV vaccination versus HBV alone (14, 38-41). Three studies indicated the effectiveness of levamisole (38-40), one study showed its negative effects (14) and another study suggested the ineffectiveness of levamisole (41). Two of them were affiliated from Turkey and three were from Iran.

**Table 1:** Summary of Literature Data: Baseline Characteristics

Study	Publication , y	Methodology	Mean Age , y	Male Gender, %	Duration of Hemodialysis , mon	Form of Levamisole Administration
**Ayli, ***et al.*	2000	RCT[Table-fn fn609]	41.4/37.4	57/60	11.8	Following each hemodialysis Session for 11 months
**Katayas,** *et al.*	2002	RCT	45/42/51/53	54/68/52/60	46/31/41/51	Following each hemodialysis session for 4 months
**Argani, ***et al.*	2006	CCT	47/45/48/41	64/54/54/54	NR	6 days before and 6 days after each vaccination
**Sali, ***et al.*	2008	Double-blinded RCT	42.9/46.6	52.6/65.6	13.2/17	After each hemodialysis session for 12 months
**Sanadgol,** *et al.*	2011	Double-blinded RCT	50.33/43.22	61.6/55.5	54.6/46.3	6 days before and 6 days after each vaccination

Abbreviations: CCT, Controlled Clinical Trials; NR, not reported; RCT, Randomized Controlled Trial

**Table 2:** Summary of Literature Data: Vaccine and Levamisole Characteristics

Study	Vaccine Administration	Levamisole Dosage, mg	Seroprotection, %	Side Effects	Levamisole Effect on Immune Response to HBV Vaccine
** Ayli ***et al*.	20 μg IM (0,1,6,9 mo)	120	13(3mo)/40(10mo)	Mild leucopenia skin rash	Increased
			20(3mo)/46(10mo)		
	40 μg IM (0,1,6,9 mo)		26(3mo)/66(10mo)		
			26(3mo)/80(10mo)		
** Katayas ***et al*.	40 μg IM (0,1,6 mo)	80	57(3mo)	Reversible mild leucopenia dermatitis	Increased
			82(3mo)		
			77(3mo)		
			15(3mo)		
** Argani ***et al*.	40 μg IM (0,1,6 mo)	100	60(1mo)/20(6mo)	Mild generalized pruritus mild abdominal pain	Increased
			70(1mo)/60(6mo)		
	20 μg ID (0,1,6 mo)		90(1mo)/80(6mo)		
	20 μg IM (0,1,6 mo)		90(1mo)/80(6mo)		
** Sali ***et al*.	40 μg IM (0,1,6 mo)	100	81.6(1mo)	No side effects	No alteration
			81.3(1mo)		
** Sanadgol ***et al*.	40 μg IM (0,1,6 mo)	100	44(0mo)/77(2mo)/77(4mo)	Severe gastrointestinal	Decreased
			55(0mo)/72(2mo)/77(4mo)	complication in one patient	

The first study took place in Turkey by Deniz Ayli, et al. Sixty hemodialysis patients were randomized into four groups compared to 15 healthy controls. The patients received IM recombinant HBV vaccine at 0, 1st, 6th and 9th month. The first group received a single dose of vaccine (20μg). Second group had double dose (40 μg) whereas group 3 received a single dose plus 120 mg levamisole and group 4 received a double dose plus 120 mg levamisole. As a result, they discovered that there was no significant difference between a double dose and a single dose vaccination considering the antibody response and there was mention of a long term application of levamisole having salient effects when added to the HBV vaccination without any serious side effects (39).

The second study included four groups of hemodialysis patients who received 40 μg of recombinant HBV vaccine at 0, 1st and 6th month. Group 1 received routine 3 doses of HBV vaccination. Group 2 received 3 doses of HBV vaccination plus 80mg levamisole whereas group 3 and 4 included unresponsive patients who did not respond to a routine vaccination program. Group 3 received 80mg levamisole plus HBV vaccine and group 4 only administered the HBV vaccine. Antibody response level in these four groups was 57%, 82%, 77% and 15%, respectively. Therefore, they had higher response level after using levamisole plus HBV vaccine (57% vs. 82%) and based on that significant difference, levamisol was recommended (77% vs. 15%) (40).

Argani et al. in 2006 evaluated the effect of levamisole and intra-dermal /intra-muscular HBV vaccination in hemodialysis patients. Forty four patients were divided into 4 equal groups. Group A received 40 μg IM vaccines. Group B received 20 μg ID vaccines. Group C received 20 μg IM vaccination plus 100 mg of levamisole and the last group received 20 μg ID vaccine plus the same dosage of levamisole. 90% of seroconversion response level was achieved in levamisole groups one month after the last dose of the HBV vaccine and lasted up to 80% for the next six months. Thus, these writers were strongly supportive of a levamisole application to HBV vaccination and proposed the ID vaccination be used routinely (38).

In another study 70 hemodialysis patients were randomized into two groups. The first group received 40 μg of HBV vaccines at 0,1st and 6th month plus 100 mg levamisole and the second group received the same vaccination protocol plus a placebo (instead of levamisole). One month after the last dose of vaccination, the seroconversion response level of Levamisole and placebo groups were 81.6%, 81.3%, respectively and there was no significant difference between these two groups (41).

Despite other studies, an interesting article in 2011 declared a lower seroconversion response level of HBV vaccination when it was accompanied by levamisole supplementation. Of 36 hemodialysis patients, 18 patients received 40 μg HBV vaccination intra-muscularly on the 0,1st and 6th month plus 100 mg of levamisole and the rest received the same amount and method of vaccination plus placebo. The seroconversion response rate was evaluated on the 0, 2nd and 4th month. Following the last dose of vaccine, the antibody response level was significantly lower immediately after HBV vaccination. However, it did not last until the 2nd and 4th months after the last dose vaccine as shown in Table 2 (14).

## 5. Discussion

There have been various strategies to improve immune response level to HBV vaccination in hemodialysis patients which has included the use of immune-modulators such as zinc (42), γ-interferon (43), granulocyte-macrophage colony-stimulating factor (GM-CSF) (44), interleukin 2 (IL2) (45), thymopentin (46), cimetidine (47), erythropoietin (44), additional immune modulators (AM3, HB-AS04) (48) and levamisole. In the current study, we performed a systemic review of the literature based on the effect of levamisole on HBV vaccination in hemodiyalysis patients. We found 2 meta-analyses related to our topic and both of them agreed that levamisole administration could increase seroprotection response rate to HBV vaccination in hemodialysis patients. It is noteworthy that we excluded these two studies because they were review articles (35, 36). Another excluded study that enrolled child patients indicated levamisole administration plus HBV vaccine therapy is not superior to vaccine alone (3). Based on our review of the literature, 3 studies suggested a significant benefit of adding levamisole to HBV vaccine to increase seroprotection levels in hemodialysis patients. Another study reported a decrease of seroprotection level and another study showed no significant difference following levamisole administration. The reason for these conflicting results may be due to the dosage of levamisole administration, the timing of administration, the dosage of the vaccine, the type of the vaccine and levamisole, the small study sample size and the host genetic background of patients (49, 50). It is incomprehensible to make a definite decision for routine administration of levamisole to HBV vaccine in hemodialysis patients given our low level of evidence nevertheless we do advocate that it is acceptable to recommend it for unresponsive patients. The availability, good tolerance and cost-effectiveness of levamisole supported this approach. Further, more comprehensive studies with larger sample sizes and the application of other types of levamisole could provide better justification for adding levamisole as an adjuvant to HBV vaccine in hemodialysis patients. However, all together, we must evaluate the combination of intramuscular and intradermal hepatitis B vaccination, implement second-generation recombinant HBV vaccination and explore the usage of other immune-modulators and adjuvants to HBV vaccine. These efforts are all needed to investigate the best approach in enhancing the response rate towards HBV vaccine in hemodialysis patients.

## References

[1] Lavanchy D (2004). Hepatitis B virus epidemiology, disease burden, treatment, and current and emerging prevention and control measures.. J Viral Hepat..

[2] Rink L, Gabriel P (2000). Zinc and the immune system. Proceeding -nutrition society of london..

[3] Wong PN, Fung TT, Mak SK, Lo KY, Tong GM, Wong Y (2005). Hepatitis B virus infection in dialysis patients.. J Gastroenterol Hepatol..

[4] Kong NC, Beran J, Kee SA, Miguel JL, Sanchez C, Bayas JM (2008). A new adjuvant improves the immune response to hepatitis B vaccine in hemodialysis patients.. Kidney Int..

[5] (2001). Recommendations for preventing transmission of infections among chronic hemodialysis patients.. MMWR Recomm Rep..

[6] Fisman DN, Agrawal D, Leder K (2002). The effect of age on immunologic response to recombinant hepatitis B vaccine: a meta-analysis.. Clin Infect Dis..

[7] Hollinger FB (1989). Factors influencing the immune response to hepatitis B vaccine, booster dose guidelines, and vaccine protocol recommendations.. Am J Med..

[8] Mahdavimazdeh M, Hosseini-Moghaddam SM, Alavian SM, Yahyazadeh H (2009). Hepatitis B Infection in hemodialysis patients in Tehran province, Iran.. Hepat Mon..

[9] Miller ER, Alter MJ, Tokars JI (1999). Protective effect of hepatitis B vaccine in chronic hemodialysis patients.. Am J Kidney Dis..

[10] Sali S (2006). HBV Vaccination in Chronic Renal Failure Patients.. Hepat Mon..

[11] Bel'eed K, Wright M, Eadington D, Farr M, Sellars L (2002). Vaccination against hepatitis B infection in patients with end stage renal disease.. Postgrad Med J..

[12] Koff RS (2002). Immunogenicity of hepatitis B vaccines: implications of immune memory.. Vaccine..

[13] Rendi-Wagner P, Kundi M, Stemberger H, Wiedermann G, Holzmann H, Hofer M (2001). Antibody-response to three recombinant hepatitis B vaccines: comparative evaluation of multicenter travel-clinic based experience.. Vaccine..

[14] Sanadgol H, Khoshnoodi M, Mashhadi MA, Forghani MS (2011). Effect of adding levamisole on seroconversion response to hepatitis B virus vaccination in hemodialysis patients: a single-center experience.. Iran J Kidney Dis..

[15] Slusarczyk Y (2002). who needs vaccination on against hepatitis B Viruses?. Vaccine..

[16] Casciato DA, McAdam LP, Kopple JD, Bluestone R, Goldberg LS, Clements PJ (1984). Immunologic abnormalities in hemodialysis patients: improvement after pyridoxine therapy.. Nephron..

[17] Eardley KS, Jones HE, Osman H, Smith SA (2002). Efficacy of the accelerated hepatitis B vaccination schedule used in haemodialysis patients post-exposure to virus: a single-centre experience.. Nephrol Dial Transplant..

[18] Rangel MC, Coronado VG, Euler GL, Strikas RA (2000). Vaccine recommendations for patients on chronic dialysis. The Advisory Committee on Immunization Practices and the American Academy of Pediatrics.. Semin Dial..

[19] Vanholder R, Van Loo A, Dhondt AM, De Smet R, Ringoir S (1996). Influence of uraemia and haemodialysis on host defence and infection.. Nephrol Dial Transplant..

[20] Laurie JA, Moertel CG, Fleming TR, Wieand HS, Leigh JE, Rubin J (1989). Surgical adjuvant therapy of large-bowel carcinoma: an evaluation of levamisole and the combination of levamisole and fluorouracil. The North Central Cancer Treatment Group and the Mayo Clinic.. J Clin Oncol..

[21] Aksoy H, Baltaci S, Turkolmez K, Seckiner I, Beduk Y (2001). Combined interferon alpha with levamisole in patients with metastatic renal cell carcinoma.. Int Urol Nephrol..

[22] Castro Garzon M, Mubita M, Kachinka L (1992). Levamisole treatment in HIV-infected Zambian children.. Lancet..

[23] De Brabander M, Vandebroek J, Wassenaar H, De Cree J, Baisier A, Demoen B (1995). Immunological alterations induced by adjuvant treatment of postoperative colon carcinoma Duke's B or C with levamisole in combination with 5-FU.. Anticancer Res..

[24] Fattovich G, Giustina G, Brollo L, Guido M, Pontisso P, Noventa F (1992). Therapy for chronic hepatitis B with lymphoblastoid interferon α and levamisole.. Hepatology..

[25] Grishchenko SV, Lavrukhina LA, Ketiladze ES, Krylov VF, Ershov FI (1984). [Results of combined therapy using levamisole for patients with influenza complicated by pneumonia].. Vopr Virusol..

[26] Holcombe RF, Li A, Stewart RM (1998). Levamisole and interleukin-2 for advanced malignancy.. Biotherapy..

[27] Katoch K (1996). Immunotherapy of leprosy.. Indian J Lepr..

[28] Link KH, Kornmann M, Staib L, Redenbacher M, Kron M, Beger HG (2005). Increase of survival benefit in advanced resectable colon cancer by extent of adjuvant treatment: results of a randomized trial comparing modulation of 5-FU + levamisole with folinic acid or with interferon-alpha.. Ann Surg..

[29] Molife R, Hancock B (2002). Adjuvant therapy of malignant melanoma.. Critic Rev Oncol Hematol..

[30] Borisova AM, Novikova TA, Glazko AV (1984). [Effect of levamisole on cellular immunity indices in chronic bronchitis and chronic pneumonia patients].. Ter Arkh..

[31] Johnkoski JA, Peterson SM, Doerr RJ, Cohen SA (1996). Levamisole regulates the proliferation of murine liver T cells through Kupffer-cell-derived cytokines.. Cancer Immunol Immunother..

[32] Modai D, Weissgarten J, Zolf R, Peller S, Averbukh Z, Kaufman S (1988). Effect of levamisole on chemotaxis of granulocytes from uremic patients.. Nephron..

[33] Demirci F, Bayraktaroglu Z, Karaoglan M, Coskun Y, Karaoglan I, Okan V (2005). Immunomodulatory effects of HBsAg vaccine and levamisole in chronic hepatitis B and hepatitis B carrier children.. Turk J Gastroenterol..

[34] Luo XL, Wang Y, Tian GS, Fu XX, Wang YY, Wei L (2004). [Therapeutic effect of levamisole plus HBV vaccine and dipyridamole on patients chronically infected by HBV with precore mutation].. Zhonghua Shi Yan He Lin Chuang Bing Du Xue Za Zhi..

[35] Alavian SM, Tabatabaei SV (2010). Effects of oral levamisole as an adjuvant to hepatitis B vaccine in adults with end-stage renal disease: a meta-analysis of controlled clinical trials.. Clin Ther..

[36] Fabrizi F, Dixit V, Messa P, Martin P (2010). Meta-analysis: levamisole improves the immune response to hepatitis B vaccine in dialysis patients.. Aliment Pharmacol Ther..

[37] Aminzadeh Z, Akhavan H, Gachkar L (2007). Source and response of Antibody to hepatitis B Vaccine in hemodialysis patients.. Hepatmon..

[38] Argani H, Akhtarishojaie E (2006). Levamizole enhances immune responsiveness to intra-dermal and intra-muscular hepatitis B vaccination in chronic hemodialysis patients.. J Immune Based Ther Vaccines..

[39] Deniz Ayli M, Ensari C, Ayli M, Mandiroglu F, Mut S (2000). Effect of oral levamisole supplementation to hepatitis B vaccination on the rate of immune response in chronic hemodialysis patients.. Nephron..

[40] Kayatas M (2002). Levamisole treatment enhances protective antibody response to hepatitis B vaccination in hemodialysis patients.. Artif Organs..

[41] Sali S, Alavian SM, Hajarizadeh B (2008). Effect of levamisole supplementation on hepatitis B virus vaccination response in hemodialysis patients.. Nephrology (Carlton)..

[42] Brodersen HP, Holtkamp W, Larbig D, Beckers B, Thiery J, Lautenschlager J (1995). Zinc supplementation and hepatitis B vaccination in chronic haemodialysis patients: a multicentre study.. Nephrol Dial Transplant..

[43] Quiroga JA, Castillo I, Porres JC, Casado S, Saez F, Gracia Martinez M (1990). Recombinant gamma-interferon as adjuvant to hepatitis B vaccine in hemodialysis patients.. Hepatology..

[44] Hassan K, Shternberg L, Alhaj M, Giron R, Reshef R, Barak M (2003). The effect of erythropoietin therapy and hemoglobin levels on the immune response to Engerix-B vaccination in chronic kidney disease.. Ren Fail..

[45] Mauri JM, Valles M (1997). Effects of recombinant interleukin-2 and revaccination for hepatitis B in previously vaccinated, non-responder, chronic uraemic patients. Collaborative Group of Girona.. Nephrol Dial Transplant..

[46] Donati D, Gastaldi L (1988). Controlled trial of thymopentin in hemodialysis patients who fail to respond to hepatitis B vaccination.. Nephron..

[47] Wang J, Su B, Ding Z, Du X, Wang B (2008). Cimetidine enhances immune response of HBV DNA vaccination via impairment of the regulatory function of regulatory T cells.. Biochem Biophys Res Commun..

[48] Perez-Garcia R, Perez-Garcia A, Verbeelen D, Bernstein ED, Villarrubia VG, Alvarez-Mon M (2002). AM3 (Inmunoferon) as an adjuvant to hepatitis B vaccination in hemodialysis patients.. Kidney Int..

[49] Renoux G (1980). The general immunopharmacology of levamisole.. Drugs..

[50] Rytel MW (1978). Can therapeutic considerations provide clues to the etiology of aphthous stomatitis and Behcet's syndrome?. J Oral Pathol..

